# Vascular Health and Cutaneous Sensation are Predictive of Upper Limb Bone Loss in People with Stroke: A 2-Year Longitudinal Study

**DOI:** 10.1007/s00223-026-01485-y

**Published:** 2026-02-12

**Authors:** Huixi Ouyang, Tiev Miller, Ling Qin, Michael Tin Cheung Ying, Vivian Wing Yin Hung, Thomas Wai Hong Leung, Julie Ye Li, Siu Ngor Fu, Raymond Chi Keung Chung, Marco Yiu Chung Pang

**Affiliations:** 1 Department of Rehabilitation Sciences, The Hong Kong Polytechnic University, 11 Yuk Choi Rd, Kowloon, Hong Kong SAR China; 2 School of Biomedical Engineering, Guangzhou Medical University, Guangzhou, China; 3 International Collaboration on Repair Discoveries, Faculty of Medicine, The University of British Columbia, Vancouver, BC Canada; 4 Division of Physical Medicine and Rehabilitation, Department of Medicine, The University of British Columbia, Vancouver, BC Canada; 5 Bone Quality and Health Centre, Center of Aging in Musculoskeletal Degeneration and Regeneration, Department of Orthopedics and Traumatology, The Chinese University of Hong Kong, Shatin, Hong Kong SAR China; 6 Department of Health Technology and Informatics, The Hong Kong Polytechnic University, Kowloon, Hong Kong SAR China; 7 Department of Medicine & Therapeutics, Faculty of Medicine, The Chinese University of Hong Kong, Shatin, Hong Kong SAR China

**Keywords:** Stroke, Bone, Upper extremity, Peripheral quantitative computed tomography

## Abstract

**Objectives:**

Secondary osteoporosis is common post-stroke. Despite the importance of fracture prevention in this population, stroke-related factors which lead to bone loss over time remain relatively understudied. This study aimed to assess changes in distal radius bone properties after stroke and examine their association with clinical assessments of stroke-related impairment.

**Design:**

Forty-five people with chronic stroke (age = 60.7 ± 7.2 years; post-stroke onset = 6.4 ± 4.2 years, 20 = women) and 45 healthy controls (age = 57.7 ± 6.3 years; 17 = women) completed this longitudinal study.

**Methods:**

Bone properties and failure load of the bilateral distal radius were measured at baseline and at 2-year follow-up using high-resolution peripheral quantitative computed tomography. Brachial artery blood flow volume and cutaneous hand sensation were measured by Doppler ultrasound and Semmes-Weinstein monofilaments, respectively.

**Results:**

Relative changes in cortical area and thickness (Cohen’s d = 0.51–0.59, 95%CI = 0.08–1.01) and estimated bone stiffness and failure load (Cohen’s d = 0.49–0.63, 95%CI = 0.07 − 1.05) were significantly greater for the stroke group than the controls (non-dominant side). On the paretic side, the decline in trabecular bone density (− 2.29%), cortical volumetric bone mineral density (vBMD) (− 0.53%), cortical thickness (− 3.61%), cortical area (− 3.45%), and increase in trabecular area (1.06%) were 2 to 4-fold greater than their corresponding precision error at follow-up. Among these, change in cortical vBMD (standardized Beta = 0.39, *p* = 0.004) and cortical thickness (standardized Beta = 0.34, *p* = 0.012) contributed most to the decrease in paretic radius failure load. Additionally, both arterial blood flow volume (ΔF = 5.31, *p* = 0.027) and light touch sensation (ΔF = 4.26, *p* = 0.046) at baseline were significant predictors of failure load decline.

**Conclusions:**

Deterioration in paretic radius bone strength was largely attributed to changes in cortical bone properties among people with chronic stroke. Autonomic dysfunction, evidenced by compromised vascular health, as well as reduced cutaneous sensation at baseline, may be associated with greater decline in radius bone strength.

**Supplementary Information:**

The online version contains supplementary material available at 10.1007/s00223-026-01485-y.

## Introduction

The wrist is the second most common site of fragility fractures among people with stroke [1]. Fractures sustained at the distal radius may require protracted hospitalization and intensive rehabilitation [2]. As people with stroke are often unable to regain pre-injury function and experience difficulties in performing routine activities of daily living [3], fracture susceptibility is a significant public health concern [4]. Previous studies using dual-energy X-ray absorptiometry (DXA) have shown that the rate of bone loss in the hemi-paretic upper limb is as high as 25% within the first year post-stroke [5]. Both DXA, the gold standard for diagnosing osteoporosis, and peripheral quantitative computed tomography (pQCT) studies have demonstrated a strong association between measures of bone integrity (e, g., bone strength index) and paretic upper limb muscle atrophy and functional impairment in people with stroke [6]. Compromised bone strength is also a major factor contributing to elevated fracture risk [7]. Thus, improving bone strength, especially in paretic upper limbs, is crucial for reducing fracture risk in this population.

According to a recent systematic review, the quality of evidence supporting the efficacy of therapeutic interventions for improving bone health post-stroke remains low, suggesting a limited understanding of the underlying clinical factors contributing to bone loss severity after stroke [3]. A growing body of evidence demonstrates a strong relationship between indices of bone strength and stroke-related deficits in motor function [5, 8–11]. A prospective study by Jørgensen & Jacobsen showed an association between paretic arm impairment severity and reduced proximal humeral bone mineral density (BMD, by DXA) during the first year after stroke [5]. Later in a study using peripheral quantitative computed tomography (pQCT), Pang et al. found that in addition to physical inactivity, paretic arm disuse and diminished vascular elasticity were associated with cortical bone thinning at the paretic radius diaphysis [11]. While, BMD and cortical thickness are important indicators of bone strength, other characteristics, such as bone geometry and microstructure (e.g., cortical porosity, trabecular thickness and trabecular number) are also key determinants and can be measured using high resolution (HR)-pQCT [12]. 

Estimated failure load, a surrogate measure of bone strength, is computed using multiple bone variables derived from HR-pQCT scans (e.g., BMD, microstructure, and geometry) [13]. In cadaver studies, estimated failure load has shown a stronger correlation with the actual mechanical failure load (i.e., the amount of mechanical loading stress equal to or exceeding the yield strength of bone) than other bone variables measured using pQCT (e.g., vBMD or cortical thickness) [14]. The ability of estimated failure load to predict fractures in older adults is also superior to that of other bone variables [15]. 

Using HR-pQCT, Miller et al. found that in addition to paretic arm motor impairment and disuse, more severe spasticity was associated with higher relative side-to-side difference in estimated failure load of the distal radius in people with chronic stroke [8]. However, this study had a cross-sectional design and could not measure bone changes over time. Temporal changes in bone strength have important implications for clinical practice and designing post-stroke rehabilitation interventions. A trial by Pang et al. showed that a comprehensive 19-week exercise program significantly increased trabecular bone content and cortical thickness of the paretic tibia in people with chronic stroke [16]. Pang and Lau also found that a structured 6-month treadmill-based exercise program was more effective for increasing tibial cortical thickness in people with chronic stroke than usual care alone [17]. Although preventative measures for reducing bone loss are often underemphasized during post-stroke rehabilitation, these results suggest that targeted interventions and physical training to enhance motor function may positively impact skeletal health in stroke-affected lower limbs. Whether similar programs can confer such benefits in the paretic upper extremity remains unknown.

A more comprehensive understanding of post-stroke changes in various bone properties is crucial for informing future intervention trials to remediate hemi-osteoporosis after stroke. Identifying clinically modifiable factors may also help guide the development of effective interventions for addressing poststroke bone loss and fracture proclivity. Therefore, longitudinal research measuring long-term changes in different bone characteristics and their associated clinical factors is warranted. To address these knowledge gaps, this prospective cohort study aimed to: (1) assess changes in bone density, geometry, microstructure and estimated failure load of the radius during the chronic stage of stroke recovery; (2) assess how changes in different bone variables contributed to changes in estimated failure load; and (3) examine the associations between changes in estimated failure load and relevant clinical assessments of stroke-related impairment.

## Materials and Methods

### Study Design

This was a 2-year longitudinal study involving individuals with chronic stroke.

### Participants

Individuals with chronic stroke were recruited from community self-help groups, existing participant databases, and the public in Hong Kong (October 2018 to September 2021). The baseline data has been published previously [8]. The inclusion criteria were diagnosis of unilateral stroke, age ≥ 18 years, stroke onset ≥ 1 year, medically stable, and Modified Rankin Scale (mRS) score ranging from 2 to 5, with higher scores indicating greater stroke-related disability. The exclusion criteria were other neurological conditions (e.g., Parkinson’s disease, intracranial tumors), receptive aphasia, recurrent stroke, pregnancy, other conditions with substantial impact on bone health (e.g., rheumatoid arthritis), taking medications for osteoporosis currently and/or prior to stroke, known diagnosis of osteoporosis prior to stroke, fragility fractures prior to stroke, metal implants in the limb to be scanned, or another serious illness (e.g., cancer). Age- and sex-matched individuals with no history of stroke were also recruited as controls through a non-probability (i.e., convenience) sampling method. Basic demographic data were obtained from participant medical records. A technician at a local bone imaging center with extensive experience in osteoporosis research performed the scan. Clinical measurements were performed by two other researchers with over five years of experience and one research assistant.

An a priori power analysis was conducted to estimate the sample size required for between-group comparisons of bone parameters according to a study using pQCT to assess the bone strength index of distal radius epiphysis in people with chronic stroke and healthy controls [18]. Based on the side × group interaction effect for this parameter, an effect size equivalent to f = 0.25 was observed. Assuming a similar effect magnitude, an alpha threshold of 0.05, power of 0.8, and an approximate attrition rate of 23% over the follow up period, it was estimated that a total of 128 participants (i.e., 64 per group) would be required. According to a study by Lam et al. [19], the difference in percent change in various HR-pQCT variables between the hemi-paretic and non-paretic sides during the late chronic stage of stroke yielded Cohen’s d values ranging from 0.25 to 1.67 (i.e., equivalent to f = 0.13–0.84). Using the smallest yielded effect size (f = 0.15) for two within-subject factors [side (non-paretic vs. hemi-paretic) and time (two assessments: baseline and 2-year follow-up)], a sample size of 90 individuals (i.e., 45 per group) would be needed to detect a significant side × time interaction effect. Subsequent analyses would involve multivariate regressions to examine associations between estimated bone failure load and other clinical variables. Previous cross-sectional studies generated R^2^ values ranging from 0.17 to 0.81 (equivalent to effect sizes f^2^ = 0.20–4.26) [18, 20–24]. Using the smallest yielded effect size (f^2^ = 0.20), an alpha of 0.05, and power of 0.80, a sample size of 42 would be needed to detect a significant association between estimated failure load and other clinical factors.

### Bone Scan Protocol

The bilateral distal radii of each participant were assessed at baseline (T1) and 2 years later (T2) for comparison. Volumetric BMD (vBMD), cross-sectional geometry, and microstructural properties of the bilateral distal radii were measured using HR-pQCT (XtremeCT II, Scanco Medical AG, Brüttisellen, Switzerland). The distal radius scan region was fixed at 9.5 mm proximal from the mid-joint line [25]. The length of the scan region spanned 9.02 mm proximally [26]. The analyses of bone images of corresponding sites were subjected to the same volume of interest (VOI) which was matched between the baseline and follow-up measurements [26]. Micro-finite element (mFE) analyses were performed using the FE-solver program included in the built-in Image Processing Language software of the HR-pQCT system (IPL-FE v1.15, Scanco Medical) to calculate the estimated failure load (N) and bone stiffness (kN/mm) [13]. Image segmentation was performed using a dual-threshold technique for voxel-by-voxel FE mesh conversion. Isotropy and elasticity material properties were tested with a linear high-friction compression model (model criteria: Young’s modulus = 10GP; bone tissue yield strain = 7000µstrain; Poisson’s ratio = 0.3) [27] to simulate an axial load of 1000 N load, comparable to sustaining a fall from standing height onto a fully outstretched arm [13]. The failure load criterion was assumed at 2% of bone tissue yield strain (i.e., 7000 µstrain) [28]. In accordance with motion artifact quality gradation guidelines, only scan images with a score ≤ 3 were retained [29]. The HR-pQCT scanner has demonstrated excellent reproducibility (small precision error). The least significant change (LSC) for each parameter is provided in Supplemental file 2.

### Other Clinical Assessments

#### Muscle Strength

A dynamometer system (Humac Norm Systems, Stoughton, Massachusetts, USA) was used to measure the isometric peak torque (N/m) of the elbow flexors [30]. The average score for three trials was used for further analysis.

*Physical activity*: The Physical Activity Scale for the Elderly (PASE) was used to assess physical activity level (score range: 0–400) [31]. Twelve questions were asked regarding the frequency and duration of leisure activities (e.g., jogging, swimming, and strength and endurance exercise), household activity, and work-related activity during the last 7 days. Higher scores indicated higher activity levels. The Chinese version of the PASE has demonstrated good test–retest reliability in older adults (ICC = 0.81) [31].

#### Sensory Function

Touch pressure threshold was assessed using Semmes–Weinstein monofilaments (SWMT) [32]. Both the dorsum of the hand (mid-length of the third proximal phalanges and mid-length of the third metacarpal bone) and the palmar side (i.e., pulp of the index and little finger, and mid-point of the thenar and hypothenar muscles) were tested [32]. A composite average score for these 6 sites based monofilament sizes was used for further analysis.

#### Vascular Health

A Doppler ultrasound system (AixPlorer, Supersonic Imagine, Aix-en-Provence, France) was used in conjunction with a linear transducer (4-15 MHz) to measure brachial artery blood flow. All participants rested in a supine position for at least 15 min before the examination commenced. The scanning of the two sides was conducted in a randomized order. The scan location was standardized at the distal third point between the coracoid process and the crease of cubital fossa of the medial side [8]. Each measurement was performed three times. Based on our pilot trial (*n* = 15), moderate inter-rater reliability (ICC = 0.59–0.60) and good to excellent intra-rater reliability (ICC = 0.82–0.93) were shown for blood flow volume of the brachial artery (Supplemental file 2).

#### Stroke-specific Assessments

The Motor Activity Log (MAL) [33] and Fugl-Meyer Motor Assessment (FMA) [34] were used to measure upper limb disuse and motor control impairment level, respectively. Wrist and finger flexor spasticity were measured using the Composite Spasticity Scale [35]. 

### Statistical Analysis

For bone outcomes and other clinical outcomes measured bilaterally, generalized estimating equation analysis (i.e., linear model type) with covariate adjustment (i.e., age and sex) was used to detect whether there was an interaction effect for time (baseline vs. 2-year follow-up), side (paretic/ non-dominant vs. non-paretic/dominant) and group factors (stroke vs. control). Next, for scale variables which showed significant time×side×group interactions, separate two-way generalized estimating equation analyses (i.e., time×side with age and sex covariate adjustment) were conducted in each group. Separate two-way generalized estimating equation analyses (i.e., group×time with age and sex covariate adjustment) were also conducted for each side.

For the stroke group data, Pearson’s correlations were used to determine the relationship between the relative change in estimated failure load on the paretic side and other clinical variables. Three separate multivariate regression models were then used to identify factors contributing to the change in estimated failure load. Variables showing significant correlation with the dependent variable (*p* < 0.1) were selected as independent predictors and entered into subsequent hierarchical multivariate regression analyses, while adjusting for potential confounding factors (i.e., demographic characteristics). As many demographic factors (e.g., age, sex, and stroke duration) were measured, these were entered into a principal component analysis (i.e., confirmatory factor reduction). Factors extracted from the principal component analysis were first entered into the regression model, followed by other clinical variables [8]. 

The first regression model involved other bone variables as the only independent variables. The second regression model included clinical variables at baseline while adjusting for potentially confounding factors (e.g., age, sex, etc.). The third regression model tested the *changes* in clinical variables with the same demographic factors. Standardized beta coefficients were used to compare the relative importance of predictors, with higher values indicating stronger effects on (or magnitude of association with) the dependent variable. F change and *p*-values from the model ANOVA were used to indicate whether adding a given predictor variable significantly increased the amount of explained variance (i.e., R²) in the regression model. A full description of all analyses performed is provided in Supplemental file 1.

## Results

### Participant Characteristics

This study recruited 64 individuals with chronic stroke and 64 healthy adults as controls. Dropout rate was similar for both groups (stroke = 28%; control = 30%), about half of which were due to the Covid-19 pandemic. Participants in the stroke group had mostly mild (mRS = 2, *n* = 31) or moderate (mRS = 3, *n* = 12) stroke-related disability. At baseline, 1 participant in the control group and 12 participants in the stroke group reported falls. At 2-year follow-up, 9 participants in the control group and 17 participants in the stroke group reported falls during the follow-up period. Two participants in stroke group sustained fractures (one paretic scapula fracture and one femural neck fracture) as well as one participant in the control group (5th phalanx fracture). Bone data for one participant from the stroke group was excluded due to motion artifact. Thus, a total of 45 individuals with stroke and 45 controls were included in the final analysis (Supplemental Fig. 1; Table 1).

### Changes in Bone Outcomes

Significant three-way interaction effects (side×time×group) were observed for most bone parameters. A significant side×time interaction was only observed for trabecular vBMD (Wald Chi-square (χ^2^) = 7.55, *p* = 0.006) in stroke group together with significant main effect of time (Wald χ^2^ = 7.55, *p* = 0.006) and side (Wald χ^2^ = 7.55, *p* = 0.006) (Table 2, Supplementary Table 2.5). Significant group×time interactions were observed for cortical thickness (Wald χ^2^ = 5.21, *p* = 0.022), estimated stiffness (Wald χ^2^ = 8.98, *p* = 0.003) and estimated failure load (Wald χ^2^ = 5.23, *p* = 0.022) for the paretic side in stroke group. Significant main effects of time and group were observed for these three parameters as well (Table 3, Supplementary Table 2.6). Relative change in total vBMD exceeded the LSC on both sides in both groups, while relative changes in cortical area and cortical thickness on the paretic side were above the LSC in the stroke group alone (Table 3).

### Predicting Change in Estimated Failure Load by Other Bone Variables

The results of the bivariate correlation analyses indicate greater decline in estimated failure load was significantly associated with a greater deterioration in total vBMD (*r* = 0.68, *p* < 0.001), cortical area (*r* = 0.61, *p* < 0.001), cortical vBMD (*r* = 0.59, *p* < 0.001), and cortical thickness (*r* = 0.53, *p* < 0.001). In contrast, a greater increase in trabecular area was significantly associated with a decline in estimated failure load (*r* = − 0.60, *p* < 0.001) (Supplemental Table 2.11).

Based on the results of the multivariate regression analysis, the decrease in estimated failure load for the paretic radius was mainly attributed to a decline in both cortical vBMD (standardized Beta = 0.39, *p* = 0.004) and cortical thickness (standardized Beta = 0.34, *p* = 0.012). These variables significantly increased the predictive accuracy of the regression model (R^2^ = 0.46), accounting for 35% and 9% of the total variance, respectively (Table 4).

### Predicting Change in Estimated Failure Load by Clinical Variables

At baseline, lower blood flow volume (*r* = 0.31, *p* = 0.036) and diminished hand light touch sensation (*r*=-0.32, *p* = 0.032) were significantly correlated with a greater decrease in estimated failure load (Supplemental Table 2.12). After adjusting for relevant demographic factors (factors 1–4: e.g., age, sex, and supplementation), both blood flow volume and hand light touch sensation at baseline significantly improved the prediction of the change in estimated failure load, accounting for 10% and 11% of the total variance, respectively (Table 5).

Changes in clinical variables over the follow-up period were then used as independent variables to predict the change in estimated failure load. The only variable showing a significant association with the change in estimated failure load was the change in physical activity (*r* = 0.27, *p* = 0.076, Supplemental Table 2.12), indicating a greater decline in physical activity was associated with greater reduction in estimated failure load. After adjusting for demographic factors, the effect of this variable on the change in estimated failure load was diminished (Table 6).

## Discussion

This study assessed longitudinal changes in distal radius bone density, geometry, and microstructure among people with chronic stroke. Compared to their healthy counterparts, a significantly greater decline in the estimated failure load of the paretic distal radius of participants with stroke was observed over the 2-year follow-up period. Among the various bone parameters assessed, the decline in cortical vBMD and cortical thickness were the most important factors contributing to the reduction in estimated failure load. Lower blood flow volume and poorer hand light touch sensation measured at baseline were independently associated greater decline in estimated failure load over the 2-year follow-up period.

### Differences in Bone Changes between Stroke and Control Groups

Compared to controls, participants in the stroke group showed greater decline in cortical area, cortical thickness, and estimated failure load on the paretic side (*cortical area*: stroke participants 3.45%, healthy controls 1.79%; *cortical thickness*: stroke participants 3.61%, healthy controls 1.72%; *estimated failure load*: stroke participants 3.32%, healthy controls 0.39%). There was a greater loss of cortical bone in the stroke group than in the control group. Due to the non-weight-bearing nature of the radius, both cortical and trabecular bone may not be subjected to the same compression stimuli as weight-bearing long bones of the lower extremities. Muscle contractions are also a major source of mechanical stress acting on bone. A decrease in tensile forces due to compromised muscle contractility following stroke may have a greater impact on cortical bone than profundal trabecular bone. Superficial cortical bone is connected to tendons via periosteum, which directly transmit mechanical stimulation during contraction. Thus, the degree of mechanical stress conferred to cortical bone is greatly diminished on the paretic side due to learned disuse after stroke [8]. The resultant bilateral disparity in cortical area and cortical thickness observed in the current study was evident among participants with stroke, but not among matched controls.

### Percent Bone Change, Failure Load, and Fracture Risk

Three specific bone properties (cortical vBMD, trabecular vBMD, and cortical thickness) were found to account for 46% of variance in estimated failure load among participants with chronic stroke. A review conducted by the Bone Microarchitecture International Consortium in 2019, which included eight prospective cohort studies, found that fracture prediction improved when these same bone properties were accounted for when compared to DXA-derived femoral neck areal BMD or fracture risk assessment tool (FRAX) scores alone [15]. 

The relationship between the decline in estimated failure load and actual fracture risk has also been investigated in previous studies [15, 36]. A recent review of four prospective HR-pQCT studies found that the radius failure load in people after fracture was 9.1% (6.7%–11.5%) lower than in people without fracture [36]. Moreover, forearm fracture risk showed a stronger association with radius failure load than other bone variables with a 5 years hazard ratio (HR) of 2.13 (1.77–2.56) per SD decrease in failure load (811 N) [15]. This suggests that for every 811 N decrease in failure load, fracture risk in older adults will increase two-fold from baseline. Among our participants with stroke, estimated failure load was already 24.6% (990 N) lower on the paretic side than the non-paretic side at baseline and further decreased by 3.3% (97 N) after 2 years. This suggests that forearm fracture risk may increase substantially during the follow-up period.

### Factors Predicting Bone Loss: Vascular Health at Baseline

Bilateral limb disparity in vascular function is substantial after stroke and may be reflective of upper limb impairment severity [37]. In this study, the decrease in paretic upper limb estimated failure load was independently associated with baseline blood flow volume. This was largely in line with evidence from a previous study by Pang et al., which found that lower vascular elasticity (ρ = 0.620, *p* = 0.004) at baseline was significantly associated with a greater decline in cortical thickness on the paretic side of the radius diaphysis at 1-year follow-up [11]. Reduced arterial blood flow volume and greater functional impairment have also been shown to be independent predictors of estimated tibial bone strength at 2-year follow-up in people with chronic stroke [9]. A large cohort study involving older adults (*n* ≈ 160,000, mean age = 58 years) also showed an association between bone quality and vascular health (i.e., arterial stiffness) [38]. As bone is a highly vascularized structure, it is plausible that compromised vascular function may have detrimental effects on bone health. Although other studies suggest that cytokines and oxidized lipids may play a role in mitigating bone loss due to osteoporosis [11, 39], the physiological mechanism underlying their association with bone strength (i.e., estimated failure load and fracture risk) remains unclear. Further research is warranted to elucidate the mechanistic link between vascular function and bone health.

### Factors Predicting Bone Loss: Hand Cutaneous Sensation at Baseline

On the paretic side, poorer hand light touch sensation at baseline was correlated with a greater decline in estimated failure load of the paretic distal radius. A recent review of physiological interactions between the nervous and skeletal systems provides cogent evidence supporting bone–nerve crosstalk [40]. Specifically, peripheral sensory nerves in the skeleton may play an essential role in promoting bone regeneration by providing trophic factors (e.g., neuropeptides: calcitonin gene-related peptide, vasoactive intestinal peptide, and substance P) [41]. Among these molecules, calcitonin gene-related peptide may potentially enhance osteoblast activity [41], whereas vasoactive intestinal peptide enables bone resorption.

Similar to skin sensory nerves, skeletal sensory nerves originate from the dorsal root ganglion next to the spinal cord which transmit pain, pressure, and other mechanical stimuli acting on bone [42]. Furthermore, according to Hilton’s law, the nerve bundles which supply the medullary cavity and articular surfaces of long bones share continuity with sensory nerves that supply the skeletal muscles and dermis [41]. It has also been shown that stroke, as a central nervous system disorder, may impair skeletal sensory nerves in addition to cutaneous sensory function. Stroke-induced loss in skeletal sensation may limit its protective role in bone formation [43, 44]. This area is understudied and awaits further research.

### Clinical Implications

Considering the association between vascular function and reduced bone strength demonstrated in this study, it is deducible that therapeutic approaches which improve vascular health may be promising interventions for maintaining bone strength and preventing bone loss in the upper limbs after stroke. A randomized controlled trial by Billinger et al. investigating the effect of an 8-week heart rate-based moderate- to high-intensity recumbent stepper aerobic exercise program for enhancing cardiovascular function in individuals with subacute stroke demonstrated significant improvements in brachial artery flow-mediated dilation for both paretic and non-paretic limbs, and increased 6-minute walk test distance [45]. A study by Kang et al. evaluating a 4-week arm ergometry aerobic exercise program for improving cardiorespiratory and motor function in people with acute stroke also showed improvements in resting heart rate, peak oxygen consumption and upper extremity motor recovery scores [46]. Whether similar aerobic exercise programs targeting the upper extremities may be useful in maintaining or enhancing bone strength in people with chronic stroke remains unknown.

Our findings also suggest that improved sensory function of the upper limb may potentially promote bone health. Evidence from a meta-analysis examining the influence of passive (e.g., peripheral nerve stimulation, thermal stimulation, and pneumatic compression) and active sensory-focused exercise therapies for enhancing somatosensory function after stroke suggests that these methods may significantly improve both sensory (e.g., light touch, joint position proprioception), and functional recovery [47]. Whether such interventions may have clinical utility in enhancing bone integrity remains unexplored. Future research is needed to explore these important areas.

### Limitations

The study period overlapped with the COVID-19 pandemic period, which contributed to the high attrition rate among participants in both groups. However, the minimum sample size required was reached (i.e., 45 people per group excluding attrition). The rate and reasons for the attrition were also similar between groups. Although sufficient for exploratory analyses, our ability to conduct more robust multivariate modelling may have been limited by the remaining sample size. Only a limited number of confounding factors influencing bone status (e.g., associated comorbidities, medication, BMI, calcium supplementation) could be accounted for in the regression models given our sample size. The non-probability sampling method used to recruit the study participants is also a potential source of bias. Future studies involving larger participant samples recruited using a probability sampling method are warranted to support the generalizability of these findings as well as examine the independent effects of individual variables on the decline in estimated failure load.

Another design limitation of the present study was the absence of biochemical markers for assessing osteogenesis (e.g., bone-specific alkaline phosphatase (BALP), osteocalcin, procollagen type 1 N-terminal propeptide (P1NP)) and osteolysis over the follow-up period (e.g., C-terminal telopeptide (CTX), N-terminal telopeptide (NTX), tartrate-resistant acid phosphatase 5b (TRACP-5b)). To date, relatively few studies involving people with stroke have incorporated both bone imaging and biomarker outcomes concurrently [48–50]. Additional longitudinal studies are needed to investigate biomarkers of bone metabolism in people with stroke in order to obtain a more comprehensive picture of these changes over time.

The Motor Activity Log used to assess upper limb disuse is influenced by individual differences in pre-stroke activity level and recall bias, particularly in the chronic stage of stroke. The development of a measure which quantifies both the frequency and intensity of physical activity, like the PASE scale but specific to the upper limb, is needed in future research. Furthermore, perceived usage frequency of the paretic arm (MAL-AOU subscale) among participants with stroke in the current study was minimal (baseline: 1.28 ± 1.40, 2-year follow-up: 1.60 ± 1.43). This suggests that the learned disuse among our participants may not be fully representative of the broader spectrum of upper limb disuse patterns and may therefore limit the generalizability of our findings. Most upper limb activities also require fine motor coordination involving the fingers. The use of domain-specific assessments of manual dexterity (e.g., Box and Block Test) are also warranted moving forward. Moreover, learned disuse of the paretic arm and hand is only one aspect of upper limb dysfunction after stroke. Impairment is also shaped by other factors such as stroke type, location and extent of the lesion, as well as the preservation of neural networks [51]. Lesions involving the corticospinal tract, motor cortex, and other regions lead to motor deficits which differ in form and severity. As the integrity of neural networks is crucial for functional recovery, future studies should incorporate a comprehensive assessment of these stroke characteristics to ensure subsequent therapeutic interventions can be tailored accordingly.

## Conclusion

In conclusion, this study found that paretic distal radius bone strength continues to decline in people with chronic stroke, which is largely due to the decrease in both cortical vBMD and thickness. Better vascular health and better hand cutaneous sensation at baseline were predictive of less decline in bone strength over the 2-year follow-up period. These findings may be useful in guiding the design of rehabilitation strategies to address post-stroke bone loss in future research trials.

**Table 1:** Demographic information

	Stroke (n = 45)		Control (n = 45)		*p*
Basic demographics					
Age (year)	60.7 (7.2)		57.7 (6.3)		0.04*
Body Mass Index (kg/m^2^)	24.2 (3.1)		23.4 (2.8)		0.05
Stroke duration (year)	6.4 (4.2)		NA		NA
Gender (Female/male, n)	20/25		17/28		0.67
Dominant hand (L/R, n)	0/45		0/45		1.00
Postmenopausal women (Yes/no, n )	18/2		15/2		0.66
Postmenopausal (women only, year)	15.2 (13.5)		10.3 (9.0)		0.30
Alcohol history (non-drinker/drinking history, n)	36/9		28/17		0.10
Smoking history (non-smoker/ smoking history, n)	35/10		33/12		0.81
Modified Rankin Scale (1/2/3, n)	2/31/12		NA		NA
Abbreviated Mental Test (Max:10)	9.33 (1.02)		9.93 (0.25)		< 0.001**
Co-morbid conditions					
Hypertension (n)	25		16		0.09
Diabetes (n)	10		6		0.41
High cholesterol (n)	15		4		0.01**
Total number of comorbidities	1.4 (1.4)		0.7 (1.0)		0.01**
Medications/supplements					
Antihypertensive agents (n)	26		12		0.01**
Anticoagulants (n)	16		0		< 0.001**
Anticonvulsive agents (n)	4		0		0.117
Hypolipidemic agents (n)	26		7		< 0.001**
Hypoglycemic agents (n)	7		4		0.522
Antidepressants (n)	5		0		0.056
Proton Pump Inhibitors (n)	19		0		< 0.001**
Calcium (n)	1		3		0.616
Vitamin D (n)	2		0		0.494
Total number of medications	4.0 (2.6)		0.8 (1.3)		< 0.001**

All values are reported as Mean (SD), unless indicated otherwise Significant between-group difference: *p<0.05, **p<0.01

*NA* not applicable, *n* number of participants

**Table 2:** Radius HR-pQCT variables for the stroke and control groups

	Stroke (*n* = 45)							Control (*n* = 45)						
	Baseline		2-Year follow up		Main effect		Interaction	Baseline		2-Year follow up		Main effect		Interaction
	*P*	NP	*P*	NP	Time: *p*	Side: *p*	Time×Side: *p*	ND	D	ND	D	*p*	*p*	Time×Side: *p*
Total vBMD (mg HA/cm^3^)	294.67 (87.53)	359.37 (67.77)	287.71 (85.46)	351.88 (67.89)	< 0.001**	< 0.001**	0.338	339.86 (63.86)	334.80 (65.14)	332.87 (63.51)	328.04 (65.53)	< 0.001**	0.111	0.820
Trabecular area (mm^2^)	186.91 (50.52)	179.48 (51.19)	188.72 (51.94)	181.15 (51.19)	< 0.001**	0.004**	0.947	195.16 (59.70)	201.54 (54.75)	196.45 (59.61)	202.68 (54.61)	< 0.001**	0.007**	0.540
Trabecular vBMD (mg HA/cm^3^)	117.61 (51.51)	149.42 (36.98)	117.00 (51.06)	149.44 (37.75)	0.035*	< 0.001**	0.006**	135.12 (37.36)	135.80 (39.57)	134.15 (37.96)	134.90 (40.12)	0.068	0.617	0.901
Trabecular number (1/mm)	1.10 (0.28)	1.27 (0.17)	1.11 (0.30)	1.28 (0.19)	0.157	< 0.001**	0.362	1.20 (0.20)	1.23 (0.20)	1.19 (0.20)	1.21 (0.19)	0.001**	0.079	0.362
Trabecular thickness (mm)	0.23 (0.02)	0.23 (0.02)	0.23 (0.02)	0.23 (0.02)	0.492	0.420	0.275	0.23 (0.02)	0.23 (0.02)	0.23 (0.02)	0.23 (0.02)	0.291	0.013*	0.287
Trabecular separation (mm)	0.95 (0.36)	0.74 (0.12)	0.94 (0.34)	0.74 (0.13)	0.913	< 0.001**	0.955	0.81 (0.19)	0.78 (0.15)	0.81 (0.18)	0.79 (0.16)	0.012*	0.183	0.073
Cortical Area (mm^2^)	55.49 (14.25)	65.57 (13.32)	53.95 (14.31)	64.02 (13.58)	< 0.001**	< 0.001**	0.494	65.86 (12.19)	67.01 (13.83)	64.70 (12.15)	65.89 (13.87)	< 0.001**	0.068	0.873
Cortical vBMD (mg HA/cm^3^)	870.70 (76.42)	914.17 (53.77)	865.68 (68.01)	906.82 (50.90)	0.019*	< 0.001**	0.556	916.61 (57.54)	913.89 (55.11)	907.18 (56.96)	904.14 (53.53)	< 0.001**	0.359	0.831
Cortical Perimeter (mm)	64.99 (8.20)	65.06 (7.89)	65.12 (8.33)	65.22 (7.96)	0.008**	0.790	0.436	66.97 (8.81)	68.19 (8.16)	67.00 (8.73)	68.41 (8.19)	0.002**	< 0.001**	0.038*
Cortical Porosity (%)	0.01 (0.01)	0.01 (0.01)	0.01 (0.01)	0.01 (0.01)	0.360	0.357	0.694	0.01 (0.01)	0.01 (0.01)	0.01 (0.01)	0.01 (0.00)	< 0.001**	0.703	0.811
Cortical Thickness (mm)	1.03 (0.26)	1.21 (0.22)	1.01 (0.27	1.18 (0.22)	< 0.001**	< 0.001**	0.426	1.18 (0.18)	1.18 (0.21)	1.16 (0.18)	1.16 (0.21)	< 0.001**	0.766	0.832
Stiffness (kN/mm)	53788.90 (17976.3)	69471.4 (17754.6)	52172.1 (17589.6)	67801.2 (17783.9)	< 0.001**	< 0.001**	0.368	66857.1 (15487.1)	69159.4 (17170.5)	66765.0 (16152.8)	68150.5 (17518.0)	0.100	0.010**	0.123
Failure load (N)	2863.13 (960.62)	3782.85 (963.03)	2793.82 (945.27)	3689.89 (964.24)	< 0.001**	< 0.001**	0.946	3625.03 (865.37)	3744.53 (932.78)	3615.93 (897.15)	3684.97 (953.10)	0.080	0.015*	0.136

two-way generalized estimating equation analyses (i.e., time×side with age and sex covariate adjustment) : *: *p* < 0.05, **: *p *<0.01

*D* Dominant Side, *HA *Hydroxyapatite, *ND *Non-dominant Side, *NP *Non-paretic Side, *P *Paretic Side, *vBMD *Volumetric Bone Mineral Density

**Table 3:** Relative change of radius HR-pQCT variables over the 2-year follow-up period

	Stroke (n = 45)		Control (n = 45)		Paretic side vs Non-dominant side			Non-paretic side vs Dominant side		
					Main effect		Interaction	Main effect		Interaction
	P	NP	ND	D	Group:* p*	Time:* p*	Time × Group: *p*	Group:* p*	Time:* p*	Time × Group: *p*
Total vBMD (mg HA/cm^3^)	− 3.3% (4.4%)†¶	− 2.1% (2.9%)†¶	− 2.1% (2.7%)†¶	− 2.1% (2.5%)†¶	0.019 *	< 0.001**	0.263	0.033 *	< 0.001**	0.706
Trabecular area (mm^2^)	1.1% (1.3%)¶	1.0% (1.2%)¶	0.7% (0.9%)¶	0.6% (1.0%)¶	0.636	< 0.001**	0.119	0.067	< 0.001**	0.151
Trabecular vBMD (mg HA/cm^3^)	− 2.3% (5.7%)¶	− 0.1% (3.0%)	− 0.8% (3.3%)	− 0.8% (3.0%)	0.055	0.003 **	0.208	0.023 *	0.234	0.209
Trabecular number (1/mm)	0.3% (5.7%)	1.1% (3.9%)	− 0.7% (3.0%)	− 1.2% (3.3%)	0.082	0.918	0.148	0.162	0.833	0.003 **
Trabecular thickness (mm)	0.1% (2.6%)	0.5% (1.7%)	− 0.3% (1.3%)	− 0.0% (0.9%)	0.876	0.633	0.481	0.193	0.137	0.104
Trabecular separation (mm)	0.6% (5.5%)	− 0.3% (2.8%)	0.6% (2.2%)	1.3% (3.2%)	0.024 *	0.485	0.972	0.095	0.052	0.016 *
Cortical Area (mm^2^)	− 3.5% (4.0%)†¶	− 2.5% (2.9%)¶	− 1.8% (2.4%)¶	− 1.7% (2.5%)¶	< 0.001**	< 0.001**	0.075	0.831	< 0.001**	0.221
Cortical vBMD (mg HA/cm^3^)	− 0.5% (3.7%)¶	− 0.8% (1.5%)¶	− 1.0% (1.3%)¶	− 1.1% (1.1%)¶	0.009 **	0.001 **	0.424	0.401	< 0.001**	0.321
Cortical Perimeter (mm)	0.2% (0.6%)	0.3% (0.5%)	0.1% (0.4%)	0.3% (0.7%)¶	0.413	0.008	0.078	0.053	< 0.001**	0.500
Cortical Porosity (%)	14.0% (63.8%)¶	20.7% (91.6%)¶	16.5% (44.7%)¶	20.9% (55.7%)¶	0.648	0.132	0.363	0.387	0.018 *	0.565
Cortical Thickness (mm)	− 3.6% (3.9%)†¶	− 2.4% (2.8%)¶	− 1.7% (2.4%)¶	− 1.8% (2.4%)¶	0.007 **	< 0.001**	0.022 *	0.240	< 0.001**	0.237
Stiffness (kN/mm)	− 3.8% (5.7%)	− 2.5% (3.9%)	− 0.3% (5.5%)	− 1.6% (3.7%)	< 0.001**	0.001 **	0.003 **	0.228	< 0.001**	0.206
Failure load (N)	− 3.3% (6.1%)	− 2.5% (4.3%)	− 0.4% (6.0%)	− 1.7% (4.0%)	< 0.001**	0.006 **	0.022*	0.165	< 0.001**	0.266

Note: mean (SD); relative change = (T2-T1)/T1, *p* < 0.05; Negative values suggest decline from baseline to follow-up

^*^: two-way generalized estimating equation analyses (i.e., time × side with age and sex covariate adjustment): *: *p* < 0.05, **: *p* < 0.01

^†^ Relative change above Least significant change (LSC, see supplemental file 2)

^¶^ Relative change above precision error

*D* Dominant Side,  *HA* Hydroxyapatite, *ND * Non-dominant Side, *NP * Non-paretic Side, *P * Paretic Side, *vBMD* Volumetric Bone Mineral Density

**Table 4:** Regression analysis: relative contribution of different bone parameters to % change in estimated failure load of the Paretic distal radius for the stroke group

	Model summary						Standardized regression coefficients		
Independent variables	*R* ^2^	Adjusted *R*^2^	ΔR^2^	ΔF	*p* (ΔF)	AIC	Beta	*p*	VIF
Cortical vBMD %change	0.35	0.34	0.35	23.26	< 0.001*	-289.41	0.45	< 0.001†	1.18
Trabecular vBMD %change	0.37	0.34	0.02	1.32	0.258	-288.80	0.04	0.735	1.15
Cortical thickness %change	0.46	0.42	0.09	6.94	0.012*	-293.83	0.34	0.012†	1.30

A standardized beta coefficient compares the strength of the effect of each independent variable to the dependent variable (the higher the value, the stronger the effect). Significant F change indicates that this variable does account for a significant amount of additional variance when it is added in the regression model%*change*, percent change, *ΔF* F-value change, *ΔR*^2^ additional predictor variance, *AIC* Akaike information criterion, *Beta* standardized regression coefficient, *R*^2^ total variance, *vBMD* volumetric bone mineral density, *VIF *variance inflation factor* *p* ≤ 0.05 Statistically significant F-value change† *p* ≤ 0.05 Statistically significant predictor; Adding %change in height to the regression model did not change the result

**Table 5:** Regression analysis: associations between % change in estimated failure load of the Paretic distal radius and other clinical variables for the stroke group at baseline (*n* = 45)

	Model Summary						Standardized Regression Coefficients		
Predictor variables	*R* ^2^	Adjusted *R*^2^	ΔR^2^	ΔF	*p* (ΔF)	AIC	Beta	*p*	VIF
Factor 1	0.01	− 0.02	0.01	0.30	0.584	-270.27	0.00	0.993	1.11
Factor 2	0.01	− 0.04	0.00	0.07	0.795	-268.34	-0.13	0.404	1.07
Factor 3	0.01	− 0.06	0.01	0.24	0.626	-266.61	-0.14	0.350	1.03
Factor 4	0.02	− 0.08	0.00	0.00	0.957	-264.61	0.01	0.959	1.03
Baseline hand sensory	0.11	0.00	0.10	4.26	0.046*	-267.27	-0.30	0.059	1.14
Baseline blood flow volume	0.22	0.10	0.11	5.31	0.027*	-271.16	0.35	0.027†	1.13

Factor 1 to 4 were generated from the principal component analysis. A standardized beta coefficient compares the strength of the effect of each individual independent variable to the dependent variable (the higher the value, the stronger the effect). Significant F change indicates that this variable does account for a significant amount of additional variance when it is added in the regression model.

Factor 1 = Age, sex, smoking history, alcohol historyFactor 2 = Calcium supplementation status; Vitamin D supplementation statusFactor 3 = Total number of medications, total number of comorbidities, BMIFactor 4 = Stroke duration

R^2^ = total variance, ΔR^2^ = additional predictor variance, ΔF = F-value change, AIC = Akaike information criterion; Beta = standardized regression coefficient, VIF = variance inflation factor; CI = confidence interval, %change = percent change

* *p* ≤ 0.05 Statistically significant F-value change

† *p* ≤ 0.05 Statistically significant predictor

Adding %change in height to the regression model did not change the result

**Table 6:** Regression analysis: associations between % change in estimated failure load of the Paretic distal radius and % change in clinical variables (*n* = 45)

	Model summary			Standardized regression coefficients		
Independent variables	*R* ^2^	ΔR^2^	ΔF	*p* (ΔF)	Beta	*p*
Factor 1	0.01	0.01	0.30	0.584	0.05	0.752
Factor 2	0.01	0.00	0.07	0.795	0.03	0.845
Factor 3	0.01	0.01	0.24	0.626	0.05	0.746
Factor 4	0.02	0.00	< 0.01	0.957	-0.04	0.798
PASE (% change)	0.08	0.06	2.73	0.107	0.26	0.107

Factor 1 = Age, gender, smoking historyFactor 2 = Calcium supplementation status; Vitamin D supplementation statusFactor 3 =Total number of medications, total number of comorbiditiesFactor 4 =stroke duration, alcohol history

*R2* total variance, *ΔR*^2^ additional predictor variance, *ΔF* F-value change, *Beta* standardized regression coefficient, *CI* confidence interval, *%change* percent change, *PASE* Physical Activity Scale for the Elderly

* *p* ≤ 0.05 Statistically significant F-value change

† *p* ≤ 0.05 Statistically significant predictor

Add %change in height to the regression model didn’t change the result

## Supplementary Information

Below is the link to the electronic supplementary material.

Supplementary Material 1

Supplementary Material 2

Supplementary Material 3
