# Supplementary figures and images for: Vascular Health and Cutaneous Sensation are Predictive of Upper Limb Bone Loss in People with Stroke: A 2-Year Longitudinal Study
Source: Calcif Tissue Int. 2026 Feb 12;117(1):25. doi: 10.1007/s00223-026-01485-y (PMC12901209; doi:10.1007/s00223-026-01485-y)

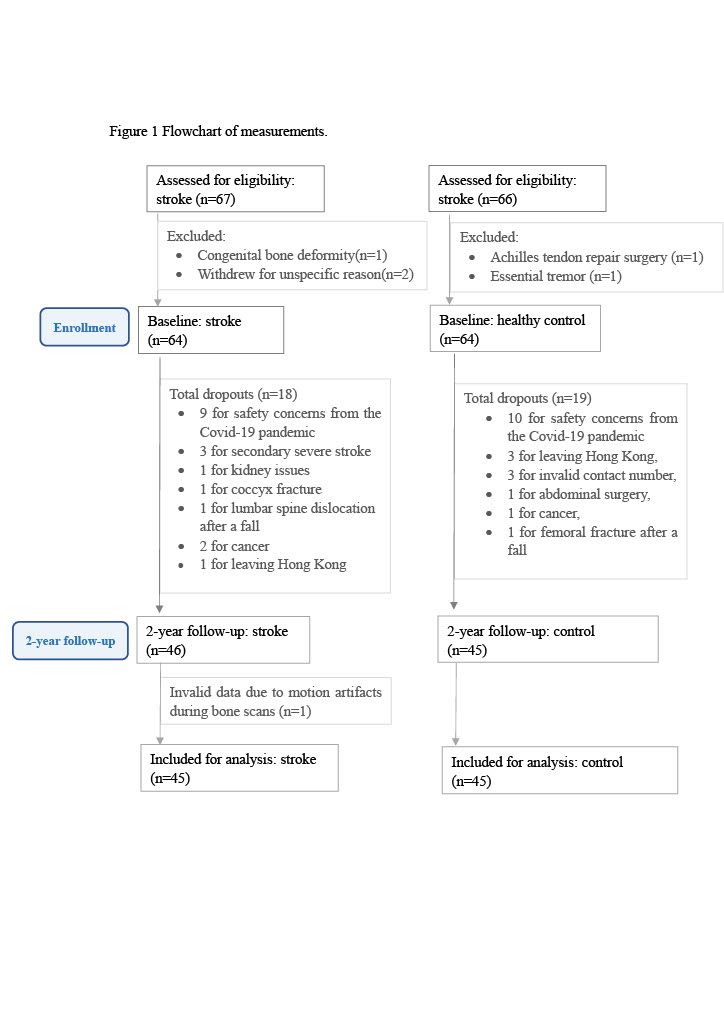

Supplement: Supplementary file 1 — Supplementary Material 1 [file 223_2026_1485_MOESM1_ESM.jpg]
