# Supplementary material for: Vascular Health and Cutaneous Sensation are Predictive of Upper Limb Bone Loss in People with Stroke: A 2-Year Longitudinal Study
Source: Calcif Tissue Int. 2026 Feb 12;117(1):25. doi: 10.1007/s00223-026-01485-y (PMC12901209; doi:10.1007/s00223-026-01485-y)
Supplement: Supplementary file 2 — Supplementary Material 2 [file 223_2026_1485_MOESM2_ESM.docx]

### **Supplemental File 2:**

**Table 2.1** Short-term reproducibility and least significant change of bone outcomes measured by HR-pQCT (n=30)

**Table 2.2** Intra-rater and inter-rater reliability of Doppler Ultrasound measurements (n=15)

**Table 2.3** Generalized estimating equation: effect of time and side and group on bone variables (n=90)

**Table 2.4** Generalized estimating equation: effect of time, side and group for clinical variables (n=90)

**Table 2.5** Generalized estimating equation: effect of time and side in two groups on bone variables (n=90)

**Table 2.6** Generalized estimating equation: effect of time and group in two sides on bone variables (n=90)

**Table 2.7** Generalized estimating equation: effect of time and side in two groups on bone variables (n=90)

**Table 2.8** Generalized estimating equation: effect of time and group in two sides on bone variables (n=90)

**Table 2.9** Changes of clinical status over the 2-year follow-up period in stroke group (n=45)

**Table 2.10** Changes of clinical variables over the 2-year follow-up period in controls (n=45)

**Table 2.11** Correlation between the change of failure load and the change of other bone variables in stroke (n=45)

**Table 2.12** Correlation between the %change of estimated failure load on the paretic side and baseline demographic and clinical variables in the stroke group (n=45)

### **Table 2.1** Short-term reproducibility and least significant change of bone outcomes measured by HR-pQCT (n=30)

| **Category** | **Bone variables** | **Precision error (CV%_RMS_)** | **Least significant change** |
| --- | --- | --- | --- |
| Bone Density | Total vBMD | 0.59% | 1.63% |
|  | Cortical vBMD | 0.46% | 1.27% |
|  | Trabecular vBMD | 0.89% | 2.47% |
| Bone Morphometry | Cortical Area | 0.94% | 2.60% |
|  | Cortical Perimeter | 0.31% | 0.86% |
|  | Cortical Thickness | 0.88% | 2.44% |
|  | Trabecular Area | 0.47% | 1.30% |
| Trabecular and Cortical Microarchitecture | Trabecular Number | 1.99% | 5.51% |
|  | Trabecular Thickness | 0.58% | 1.61% |
|  | Trabecular Separation | 1.77% | 4.90% |
|  | Cortical Porosity | 12.46% | 34.51% |

Note: The coefficient of variation (CV) was calculated as the standard deviation (SD) of the two repeated measurements of each participant divided by their means. The short-term precision error was calculated as the root-mean-square average of the CV (CV%RMS) for each participant. The 95% confidence least significant change (LSC) values were calculated as 2.77 × the precision error.

### **Table 2.2** Intra-rater and inter-rater reliability of Doppler Ultrasound measurements (n=15)

| **Model** | **Rater** | **Peak systolic velocity (cm/s)** | | **Diameter (mm)** | | **Peak volume flow (cc/min)** | |
| --- | --- | --- | --- | --- | --- | --- | --- |
|  |  | **P** | **NP** | **P** | **NP** | **P** | **NP** |
| Inter-rater (ICC 2,3) |  | 0.87 | 0.88 | 0.96 | 0.92 | 0.59 | 0.60 |
| Intra-rater (ICC 3,3) | Rater 1 | 0.97 | 0.92 | 0.99 | 0.97 | 0.93 | 0.82 |
|  | Rater 2 | 0.95 | 0.96 | 0.97 | 0.98 | 0.86 | 0.93 |

Note: P: paretic side; NP: non-paretic side.

### **Table 2.3** Generalized estimating equation: effect of time and side and group on bone variables (n=90)

|  | **Main Effect** | | | | | |  | **Interaction Effects** | | | | |
| --- | --- | --- | --- | --- | --- | --- | --- | --- | --- | --- | --- | --- |
|  | **Group** | | **Time** | | **Side** | | **Time*Group** | | **Side*Time** | | **Side*Time*Group** | |
|  | Wald χ^2^ | *p* | Wald χ^2^ | *p* | Wald χ^2^ | *p* | Wald χ^2^ | *p* | Wald χ^2^ | *p* | Wald χ^2^ | *p* |
| Total vBMD (mg HA/cm3) | 0.06 | 0.804 | 61.34 | <0.001** | 90.85 | <0.001** | 0.53 | 0.467 | 0.88 | 0.349 | 123.44 | <0.001** |
| Trabecular area (mm2) | 1.38 | 0.241 | 59.82 | <0.001** | 0.10 | 0.750 | 1.41 | 0.235 | 0.05 | 0.832 | 15.90 | <0.001** |
| Trabecular vBMD (mg HA/cm3) | 0.00 | 0.992 | 7.89 | 0.005** | 66.93 | <0.001** | 0.18 | 0.668 | 6.15 | 0.013* | 67.82 | <0.001** |
| Trabecular number (1/mm) | 0.11 | 0.739 | 0.02 | 0.891 | 37.90 | <0.001** | 7.14 | 0.008** | 0.03 | 0.866 | 23.47 | <0.001** |
| Trabecular thickness (mm) | 0.30 | 0.584 | 0.00 | 0.950 | 0.39 | 0.532 | 1.25 | 0.264 | 2.04 | 0.154 | 5.44 | 0.066 |
| Trabecular separation (mm) | 0.77 | 0.380 | 0.80 | 0.370 | 25.33 | <0.001** | 1.03 | 0.309 | 0.16 | 0.689 | 19.95 | <0.001** |
| Cortical Area (mm2) | 3.92 | 0.048* | 75.00 | <0.001** | 76.61 | <0.001** | 2.65 | 0.103 | 0.43 | 0.513 | 48.91 | <0.001** |
| Cortical vBMD (mg HA/cm3) | 1.06 | 0.302 | 27.50 | <0.001** | 41.78 | <0.001** | 1.19 | 0.276 | 0.39 | 0.532 | 54.81 | <0.001** |
| Cortical Perimeter (mm) | 1.96 | 0.162 | 16.44 | <0.001** | 8.26 | 0.004** | 0.01 | 0.913 | 4.45 | 0.035* | 7.80 | 0.020 |
| Cortical Porosity (%) | 0.57 | 0.450 | 6.21 | 0.013* | 0.99 | 0.321 | 0.72 | 0.398 | 0.20 | 0.652 | 0.47 | 0.792 |
| Cortical Thickness (mm) | 0.71 | 0.400 | 79.63 | <0.001** | 50.26 | <0.001** | 3.19 | 0.074 | 0.32 | 0.570 | 55.07 | <0.001** |
| Stiffness (kN/mm) | 3.72 | 0.054 | 25.82 | <0.001** | 104.63 | <0.001** | 7.64 | 0.006** | 0.52 | 0.469 | 66.88 | <0.001** |
| Failure load (N) | 4.10 | 0.043* | 21.65 | <0.001** | 109.26 | <0.001** | 4.68 | 0.031* | 1.38 | 0.240 | 72.86 | <0.001** |

Note: *: *p* < 0.05, **: *p* < 0.01.

### **Table 2.4** Generalized estimating equation: effect of time, side and group for clinical variables (n=90)

|  | **Main Effect** | | | | | | | | **Interaction Effects** | | | | | | | | |
| --- | --- | --- | --- | --- | --- | --- | --- | --- | --- | --- | --- | --- | --- | --- | --- | --- | --- |
|  | **Group** | | **Time** | | | | **Side** | | **Time*Group** | | | **Side*Time** | | | | **Side*Time*Group** | |
|  | **Wald χ^2^** | ***p*** | | **Wald χ^2^** | ***p*** | **Wald χ^2^** | | ***p*** | | **Wald χ^2^** | ***p*** | | **Wald χ^2^** | ***p*** | **Wald χ^2^** | | ***p*** |
| Biceps muscle strength (Nm) | 21.75 | <0.001** | | 56.76 | <0.001** | 116.31 | | <0.001** | | 35.91 | <0.001** | | 0.25 | 0.620 | 110.11 | | <0.001** |
| Biceps stiffness (N/m) | 21.27 | <0.001** | | 30.77 | <0.001** | 30.83 | | <0.001** | | 5.38 | 0.020* | | 0.07 | 0.790 | 25.71 | | <0.001** |
| PSV-Brachial artery (cm/s) | 0.10 | 0.748 | | 2.68 | 0.102 | 1.75 | | 0.186 | | 0.02 | 0.888 | | 1.57 | 0.211 | 9.07 | | 0.011* |
| PVF-Brachial artery (cc/min) | 0.06 | 0.806 | | 25.91 | <0.001** | 3.24 | | 0.072 | | 12.13 | <0.001** | | 9.58 | 0.002** | 0.45 | | 0.797 |
| Diameter-Brachial artery (cm) | 0.60 | 0.441 | | 93.40 | <0.001** | 92.79 | | <0.001** | | 0.10 | 0.758 | | 0.43 | 0.513 | 41.16 | | <0.001** |
| Hand sensation | 38.67 | <0.001** | | 19.36 | <0.001** | 25.10 | | <0.001** | | 3.72 | 0.054 | | 0.17 | 0.679 | 30.43 | | <0.001** |

Note: *: *p* < 0.05, **: *p* < 0.01.

### **Table 2.5** Generalized estimating equation: effect of time and side in two groups on bone variables (n=90)

|  | **Stroke Group (n=45)** | | | | | | **Control Group (n=45)** | | | | | |
| --- | --- | --- | --- | --- | --- | --- | --- | --- | --- | --- | --- | --- |
|  | **Main Effect** | | | | **Interaction Effects** | | **Main Effect** | | | | **Interaction Effects** | |
|  | **Time** | | **Side** | | **Side*Time** | | **Time** | | **Side** | | **Side*Time** | |
|  | **Wald χ^2^** | ***p*** | **Wald χ^2^** | ***p*** | **Wald χ^2^** | ***p*** | **Wald χ^2^** | ***p*** | **Wald χ^2^** | ***p*** | **Wald χ^2^** | ***p*** |
| Total vBMD (mg HA/cm3) | 29.43 | <0.001** | 139.50 | <0.001** | 0.92 | 0.338 | 33.43 | <0.001** | 2.54 | 0.111 | 0.05 | 0.820 |
| Trabecular area (mm2) | 32.81 | <0.001** | 8.33 | 0.004** | 0.00 | 0.947 | 27.46 | <0.001** | 7.28 | 0.007** | 0.38 | 0.540 |
| Trabecular vBMD (mg HA/cm3) | 4.46 | 0.035* | 72.65 | <0.001** | 7.55 | 0.006** | 3.33 | 0.068 | 0.25 | 0.617 | 0.02 | 0.901 |
| Trabecular number (1/mm) | 2.00 | 0.157 | 35.39 | <0.001** | 0.83 | 0.362 | 11.32 | 0.001** | 3.08 | 0.079 | 0.83 | 0.362 |
| Trabecular thickness (mm) | 0.47 | 0.492 | 0.65 | 0.420 | 1.19 | 0.275 | 1.12 | 0.291 | 6.18 | 0.013* | 1.14 | 0.287 |
| Trabecular separation (mm) | 0.01 | 0.913 | 23.73 | <0.001** | 0.00 | 0.955 | 6.28 | 0.012* | 1.77 | 0.183 | 3.22 | 0.073 |
| Cortical Area (mm2) | 48.28 | <0.001** | 81.54 | <0.001** | 0.47 | 0.494 | 27.42 | <0.001** | 3.33 | 0.068 | 0.03 | 0.873 |
| Cortical vBMD (mg HA/cm3) | 5.52 | 0.019* | 65.11 | <0.001** | 0.35 | 0.556 | 46.33 | <0.001** | 0.84 | 0.359 | 0.05 | 0.831 |
| Cortical Perimeter (mm) | 7.10 | 0.008** | 0.07 | 0.790 | 0.61 | 0.436 | 10.01 | 0.002** | 15.27 | <0.001** | 4.33 | 0.038* |
| Cortical Porosity (%) | 0.84 | 0.360 | 0.85 | 0.357 | 0.15 | 0.694 | 13.65 | <0.001** | 0.15 | 0.703 | 0.06 | 0.811 |
| Cortical Thickness (mm) | 47.90 | <0.001** | 75.40 | <0.001** | 0.63 | 0.426 | 31.87 | <0.001** | 0.09 | 0.766 | 0.05 | 0.832 |
| Stiffness (kN/mm) | 30.43 | <0.001** | 101.12 | <0.001** | 0.81 | 0.368 | 2.71 | 0.100 | 6.67 | 0.010** | 2.38 | 0.123 |
| Failure load (N) | 23.42 | <0.001** | 107.03 | <0.001** | 0.01 | 0.946 | 3.06 | 0.080 | 5.87 | 0.015* | 2.22 | 0.136 |

Note: *: *p* < 0.05, **: *p* < 0.01.

### **Table 2.6** Generalized estimating equation: effect of time and group in two sides on bone variables (n=90)

|  | **Paretic/Non-dominant Side** | | | | | | **Non-paretic/Dominant Side** | | | | | |
| --- | --- | --- | --- | --- | --- | --- | --- | --- | --- | --- | --- | --- |
|  | **Main Effect** | | | | **Interaction Effects** | | **Main Effect** | | | | **Interaction Effects** | |
|  | **Group** | | **Time** | | **Group*Time** | | **Group** | | **Time** | | **Group*Time** | |
|  | **Wald χ^2^** | ***p*** | **Wald χ^2^** | ***p*** | **Wald χ^2^** | ***p*** | **Wald χ^2^** | ***p*** | **Wald χ^2^** | ***p*** | **Wald χ^2^** | ***p*** |
| Total vBMD (mg HA/cm3) | 5.49 | 0.019 * | 49.89 | <0.001** | 1.25 | 0.263 | 4.53 | 0.033 * | 54.90 | <0.001** | 0.14 | 0.706 |
| Trabecular area (mm2) | 0.22 | 0.636 | 56.99 | <0.001** | 2.43 | 0.119 | 3.35 | 0.067 | 57.74 | <0.001** | 2.07 | 0.151 |
| Trabecular vBMD (mg HA/cm3) | 3.69 | 0.055 | 9.02 | 0.003 ** | 1.59 | 0.208 | 5.14 | 0.023 * | 1.42 | 0.234 | 1.58 | 0.209 |
| Trabecular number (1/mm) | 3.02 | 0.082 | 0.01 | 0.918 | 2.10 | 0.148 | 1.95 | 0.162 | 0.05 | 0.833 | 9.12 | 0.003 ** |
| Trabecular thickness (mm) | 0.02 | 0.876 | 0.23 | 0.633 | 0.50 | 0.481 | 1.70 | 0.193 | 2.21 | 0.137 | 2.65 | 0.104 |
| Trabecular separation (mm) | 5.12 | 0.024 * | 0.49 | 0.485 | 0.00 | 0.972 | 2.79 | 0.095 | 3.79 | 0.052 | 5.82 | 0.016 * |
| Cortical Area (mm2) | 15.22 | <0.001** | 57.52 | <0.001** | 3.17 | 0.075 | 0.05 | 0.831 | 57.45 | <0.001** | 1.50 | 0.221 |
| Cortical vBMD (mg HA/cm3) | 6.83 | 0.009 ** | 11.72 | 0.001 ** | 0.64 | 0.424 | 0.70 | 0.401 | 49.91 | <0.001** | 0.99 | 0.321 |
| Cortical Perimeter (mm) | 0.67 | 0.413 | 7.05 | 0.008 | 3.10 | 0.078 | 3.75 | 0.053 | 18.84 | <0.001** | 0.45 | 0.500 |
| Cortical Porosity (%) | 0.21 | 0.648 | 2.27 | 0.132 | 0.83 | 0.363 | 0.75 | 0.387 | 5.58 | 0.018 * | 0.33 | 0.565 |
| Cortical Thickness (mm) | 7.21 | 0.007 ** | 66.88 | <0.001** | 5.21 | 0.022 * | 1.38 | 0.240 | 61.31 | <0.001** | 1.40 | 0.237 |
| Stiffness (kN/mm) | 19.39 | <0.001** | 10.71 | 0.001 ** | 8.98 | 0.003 ** | 1.45 | 0.228 | 26.26 | <0.001** | 1.60 | 0.206 |
| Failure load (N) | 21.28 | <0.001** | 7.63 | 0.006 ** | 5.23 | 0.022* | 1.93 | 0.165 | 25.76 | <0.001** | 1.24 | 0.266 |

Note: *: *p* < 0.05, **: *p* < 0.01.

### **Table 2.7** Generalized estimating equation: effect of time and side in two groups on bone variables (n=90)

|  | **Stroke Group** | | | | | | **Control Group** | | | | | |
| --- | --- | --- | --- | --- | --- | --- | --- | --- | --- | --- | --- | --- |
|  | **Main Effect** | | | | **Interaction Effects** | | **Main Effect** | | | | **Interaction Effects** | |
|  | **Time** | | **Side** | | **Side*Time** | | **Time** | | **Side** | | **Side*Time** | |
|  | **Wald χ^2^** | ***p*** | **Wald χ^2^** | ***p*** | **Wald χ^2^** | ***p*** | **Wald χ^2^** | ***p*** | **Wald χ^2^** | ***p*** | **Wald χ^2^** | ***p*** |
| Biceps muscle strength (Nm) | 1.15 | 0.283 | 127.44 | <0.001** | 1.54 | 0.214 | 94.39 | <0.001** | 0.90 | 0.342 | 0.85 | 0.357 |
| Biceps stiffness (N/m) | 24.13 | <0.001** | 40.94 | <0.001** | 0.32 | 0.569 | 7.26 | 0.007** | 0.23 | 0.633 | 2.18 | 0.140 |
| PSV-Brachial artery (cm/s) | 0.94 | 0.333 | 1.11 | 0.293 | 0.07 | 0.789 | 1.96 | 0.161 | 9.29 | 0.002** | 2.24 | 0.134 |
| PVF-Brachial artery (cc/min) | 1.77 | 0.184 | 0.69 | 0.406 | 3.18 | 0.075 | 28.96 | <0.001** | 3.28 | 0.07 | 7.19 | 0.007** |
| Diameter-Brachial artery (cm) | 30.11 | <0.001** | 80.60 | <0.001** | 3.37 | 0.066 | 91.01 | <0.001** | 13.84 | <0.001** | 2.24 | 0.134 |
| Hand sensation | 11.04 | 0.001** | 27.54 | <0.001** | 0.16 | 0.686 | 16.39 | <0.001** | 2.65 | 0.104 | 0.01 | 0.931 |

Note: *: *p* < 0.05, **: *p* < 0.01.

### **Table 2.8** Generalized estimating equation: effect of time and group in two sides on bone variables (n=90)

|  | **Paretic /Non-dominant Side** | | | | | | **Non-paretic /Dominant Side** | | | | | |
| --- | --- | --- | --- | --- | --- | --- | --- | --- | --- | --- | --- | --- |
|  | **Main Effect** | | | | **Interaction Effects** | | **Main Effect** | | | | **Interaction Effects** | |
|  | **Group** | | **Time** | | **Group*Time** | | **Group** | | **Time** | | **Group*Time** | |
|  | **Wald χ^2^** | ***p*** | **Wald χ^2^** | ***p*** | **Wald χ^2^** | ***p*** | **Wald χ^2^** | ***p*** | **Wald χ^2^** | ***p*** | **Wald χ^2^** | ***p*** |
| Biceps muscle strength (Nm) | 74.29 | <0.001** | 31.98 | <0.001** | 32.66 | <0.001** | 0.13 | 0.715 | 37.82 | <0.001** | 13.68 | <0.001** |
| Biceps stiffness (N/m) | 42.93 | <0.001** | 20.39 | <0.001** | 6.69 | 0.010** | 1.96 | 0.161 | 24.51 | <0.001** | 1.82 | 0.177 |
| PSV-Brachial artery (cm/s) | 0.43 | 0.510 | 3.97 | 0.046* | 0.36 | 0.546 | 1.72 | 0.190 | 0.38 | 0.536 | 0.17 | 0.681 |
| PVF-Brachial artery (cc/min) | 0.23 | 0.629 | 29.58 | <0.001** | 8.73 | 0.003** | 0.00 | 0.960 | 10.90 | 0.001** | 10.22 | 0.001** |
| Diameter-Brachial artery (cm) | 1.76 | 0.185 | 79.22 | <0.001** | 1.99 | 0.158 | 7.02 | 0.008** | 71.68 | <0.001** | 0.54 | 0.464 |
| Hand sensation | 40.50 | <0.001** | 10.87 | 0.001** | 2.33 | 0.127 | 0.89 | 0.346 | 24.05 | <0.001** | 4.07 | 0.044* |

Note: *: *p* < 0.05, **: *p* < 0.01.

### **Table 2.9** Changes of clinical status over the 2-year follow-up period in stroke group (n=45)

|  | **Baseline** | **2-year follow-up** | ***p*** |
| --- | --- | --- | --- |
| Biceps muscle strength (Paretic Side, Nm) | 17.42±8.86 | 17.38±9.39 | 0.969 |
| Biceps muscle strength (Non-Paretic Side, Nm) | 29.14±10.82 | 30.73±11.06 | 0.118 |
| Composite Spasticity Scale - Upper Limb Total (Max: 16) | 8.67±2.41 | 8.20±2.16 | 0.102 |
| MAL- Amount of Use (Frequency, Max: 5) | 1.28±1.40 | 1.60±1.43 | <0.001** |
| MAL- Quality of Movement (Quality, Max: 5) | 1.40±1.43 | 1.66±1.43 | <0.001** |
| Fugl-Meyer Assessment - Upper Limb (Max: 66) | 34.46±18.87 | 40.09±16.03 | 0.005** |
| Bicep stiffness (Paretic Side, N/m) | 229.22±30.40 | 255.31±40.50 | <0.001** |
| Bicep stiffness (Non-Paretic Side, N/m) | 206.89±33.21 | 229.29±31.42 | <0.001** |
| Peak systolic velocity (Paretic Side, cm/s) | 76.67±15.00 | 78.96±18.58 | NA |
| Blood flow volume (Paretic Side, cc/min) | 42.82±24.99 | 52.85±35.39 | **0.055** |
| Arterial diameter (Paretic Side, cm) | 0.33±0.06 | 0.36±0.07 | <0.001** |
| Peak systolic velocity (Non-Paretic Arm, cm/s) | 75.50±16.36 | 77.08±17.73 | 0.506 |
| Blood flow volume (Non-Paretic Arm, cc/min) | 50.19±23.72 | 50.54±26.11 | 0.938 |
| Arterial diameter (Non-Paretic Arm, cm) | 0.38±0.06 | 0.42±0.07 | <0.001** |
| Hand light touch sensation (Paretic Side, Max: 6.65) | 3.81±1.49 | 4.27±1.45 | 0.032* |
| Hand light touch sensation (Non-Paretic Side, Max: 6.65) | 2.78±0.61 | 3.18±0.41 | 0.001** |
| Physical Activity Scale for the Elderly (Max: 400) | 120.91±79.16 | 101.55±76.97 | **0.061** |

Note: Mean±SD. *: *p* < 0.05, **: *p* < 0.01. *p* value in bold: marginally significant.

### **Table 2.10** Changes of clinical variables over the 2-year follow-up period in controls (n=45)

|  | **Baseline** | **2-year follow-up** | ***p*** |
| --- | --- | --- | --- |
| Biceps muscle strength (non-Dominant, Nm) | 26.99±9.75 | 34.22±10.56 | <0.001** |
| Biceps muscle strength (Dominant, Nm) | 27.85±10.17 | 34.24±11.42 | <0.001** |
| Bicep stiffness (non-Dominant, N/m) | 205.11±23.99 | 212.20±22.67 | 0.080 |
| Bicep stiffness (Dominant, N/m) | 201.02±25.18 | 213.82±28.31 | 0.006** |
| Peak systolic velocity (non-Dominant, cm/s) | 72.38±17.19 | 76.66±16.48 | 0.062 |
| Blood flow volume (non-Dominant, cc/min) | 30.59±21.97 | 64.44±39.30 | <0.001** |
| Arterial diameter (non-Dominant, cm) | 0.34±0.06 | 0.38±0.07 | <0.001** |
| Peak systolic velocity (Dominant Arm, cm/s) | 78.51±15.07 | 78.83±15.73 | NA |
| Blood flow volume (Dominant Arm, cc/min) | 41.41±23.35 | 62.86±37.03 | <0.001** |
| Arterial diameter (Dominant Arm, cm) | 0.35±0.06 | 0.39±0.07 | <0.001** |
| Hand light touch sensation (non-Dominant, Max:6.65) | 2.76±0.25 | 2.93±0.35 | <0.001** |
| Hand light touch sensation (Dominant, Max:6.65) | 2.80±0.30 | 2.97±0.38 | 0.001** |
| Physical Activity Scale for the Elderly (Max:400) | 148.26±82.16 | 147.43±72.06 | 0.928 |

Note: Mean±SD. *: *p* < 0.05, **: *p* < 0.01.

### **Table 2.11** Correlation between the % change in paretic radius estimated failure load and the % change in other paretic radius bone variables in the stroke group (n=45)

|  | **Estimated failure load (% change)** | |
| --- | --- | --- |
|  | **Pearson’s r** | *p* |
| Total vBMD (% change) | 0.68 | <0.001** |
| Cortical vBMD (% change) | 0.59 | <0.001** |
| Trabecular vBMD (% change) | 0.25 | 0.100 |
| Cortical area (% change) | 0.61 | <0.001** |
| Trabecular area (% change) | -0.60 | <0.001** |
| Cortical thickness (% change) | 0.53 | <0.001** |
| Cortical perimeter (% change) | 0.02 | 0.876 |

Note: **: *p* < 0.001.

### **Table 2.12** Correlation between % change in paretic side estimated failure load and baseline demographic, clinical variables and % change clinical variables in the stroke group (n=45)

|  | **Estimated failure load: %change** | |
| --- | --- | --- |
|  | r | *p* |
| **Demographic information** | 0.18 | 0.243 |
| Age (years) | 0.18 | 0.251 |
| Stroke Duration (years) | -0.15 | 0.323 |
| Total number of medications | -0.11 | 0.493 |
| Total number of comorbidities | -0.01 | 0.977 |
| BMI | -0.03 | 0.863 |
| Sex | 0.02 | 0.919 |
| Calcium Supplementation | -0.15 | 0.323 |
| Vitamin D Supplementation | -0.16 | 0.304 |
| Smoking history | 0.07 | 0.670 |
| Alcohol history | 0.18 | 0.243 |
| Hypertension | -0.29 | **0.050** |
| Hyperlipidemia | -0.06 | 0.695 |
| Diabetes Mellitus | 0.11 | 0.488 |
| **Baseline clinical variables** |  |  |
| Physical Activity Scale for Elderly | 0.038 | 0.805 |
| Biceps strength | 0.17 | 0.274 |
| Composite Spasticity Scale- upper limb | -0.013 | 0.932 |
| Fugl-Meyer Assessment-upper limb | -0.03 | 0.835 |
| Hand sensation | -0.32 | **0.032*** |
| Blood flow volume | 0.31 | **0.036*** |
| Artery diameter | 0.13 | 0.394 |
| Bicep stiffness | 0.20 | 0.184 |
| MAL frequency | 0.02 | 0.882 |
| MAL quality | 0.07 | 0.643 |
| **The relative change of clinical variables** | | |
| MAL frequency-% change | <0.01 | 0.979 |
| MAL quality-% change | -0.08 | 0.595 |
| Physical activity for elderly-% change | -0.27 | **0.076** |
| Fugl-Meyer Assessment-upper limb-% change | 0.03 | 0.823 |
| Hand sensation- absolute change^a^ | -0.22 | 0.152 |
| Blood flow volume-% change | 0.23 | 0.124 |
| Artery diameter-% change | 0.08 | 0.588 |
| Bicep stiffness-% change | -0.16 | 0.288 |

Note: **p*<0.05; *p* value in **bold**: p<0.1; % change = (T2-T1)/T1; ^a^absolute change = T2-T1.

Abbreviations: BMI = Body Mass Index, MAL = Motor Activity Log
