# Supplementary material for: Vascular Health and Cutaneous Sensation are Predictive of Upper Limb Bone Loss in People with Stroke: A 2-Year Longitudinal Study
Source: Calcif Tissue Int. 2026 Feb 12;117(1):25. doi: 10.1007/s00223-026-01485-y (PMC12901209; doi:10.1007/s00223-026-01485-y)
Supplement: Supplementary file 3 — Supplementary Material 3 [file 223_2026_1485_MOESM3_ESM.docx]

# **Supplemental File 1:**

**Supplementary Appendix 1.1**: Bone scan protocol and imaging methods

**Supplementary Appendix 1.2**: Clinical assessments

**Supplementary Appendix 1.3:** Ultrasound protocol for brachial artery measures

**Supplementary Appendix 1.4**: Statistical analyses

## **Supplementary Appendix 1.1**: Bone scan protocol and imaging methods

The bilateral distal radii of each participant were assessed twice: at baseline and 2 years later. This time frame was chosen for two reasons. First, two previous longitudinal studies showed that while bone mass of the hemi-paretic and non-paretic sides were substantially different in participants who were well into the chronic stage of stroke recovery (onset > 1 year), the temporal changes in bone variables within a 1-year period in these participants were modest.^1,2^ Thus, the present study aimed to evaluate the changes in bone status during the chronic stage of post-stroke recovery with a 2-year assessment interval. Given that the effective radiation dose for each HR-pQCT scan is only 3–4 µSv, the total effective radiation dose during the 2-year follow-up period would be approximately 12–16 µSv (i.e., 4 scans: 2 bone sites × 2 time points), which is similar to that received during 1–1.3-h flight. The protocol used in the current study was considered safe and posed minimal risk.

*Imaging and image registration:* Volumetric BMD (vBMD), cross-sectional geometry, and microstructural properties of the bilateral distal radii were measured using HR-pQCT (XtremeCT II, Scanco Medical AG, Brüttisellen, Switzerland). The distal radius scan region was fixed at 9.5 mm proximal from the mid-joint line.^3^ The length of the scan region spanned 9.02 mm proximally, which was equivalent to a stack of 110 slices.^4^ The analyses of bone images of corresponding sites were subjected to the same volume of interest (VOI) which matched between baseline and 2-year follow-up measurements. A software program provided by the HR-pQCT manufacturer was used to identify the same region by matching the corresponding images with their total cross-sectional area. Image analyses were only conducted for the bone volume common to the measurements at the two time points.^4^

*Image analysis:* Analyses of 3D scan data were conducted using the Image Processing Language software (IPL v5.08b, Scanco Medical AG). First, bone images were analyzed using a standard (default) protocol.^5^ Next, extended cortical analyses were conducted using a cortical compartment segmentation technique adapted from the study by Buie et al.^6^ (µCT Evaluation v6.0, Scanco Medical AG).^7^ This was done to achieve improved accuracy in the analyses of very porous or thin cortical shells.

*Micro-finite element (mFE) analyses:* All mFE analyses were performed using the FE-solver included in the built-in Image Processing Language software of HR-pQCT (IPL-FE v1.15, Scanco Medical).^8^ A special peeling algorithm specifying a minimum cortical thickness of six voxels was used to identify cortical and trabecular bone tissue. mFE analyses were performed by converting the binary image data into a mesh of isotropic brick elements. For all elements, a Poisson’s ratio of 0.3 was specified. Elements representing cortical and trabecular bone were both assigned a Young’s modulus of 10 GPa.^8^ A uniaxial compression test with a 1000 N load was performed with an applied strain of 1%. Calculation of estimated failure load (N) was based on the assumption that bone failure occurred if >2% of the elements were strained beyond 0.7% strain.^9^ Whole-bone stiffness (kN/mm) was also calculated to serve as an estimate of apparent bone strength.

The intra-rater reliability of these parameters was good (linear weighted kappa = 0.855).^10^ Our HR-pQCT scanner had excellent reproducibility. The coefficient of variation (CV) was calculated as the standard deviation (SD) of the two repeated measurements for each participant divided by their mean. The short-term precision error was calculated as the root-mean-square average of the CV (CV%_RMS_) for each participant. According to our unpublished data from 32 individuals, the short-term reproducibility of the vBMD parameter, expressed as the CV%_RMS_, ranged from 0.46% to 0.89%, and the short-term reproducibility of the microarchitectural parameters (excluding cortical porosity) ranged from 0.88% to 1.99%. The 95% confidence least significant change (LSC) values (calculated as 2.77 × the precision error) are also provided in Table 3-1 below.

Eq. (A.1)
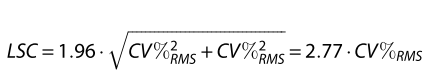


## **Supplementary Appendix 1.2**: Clinical assessments

Participants in both groups were assessed for the following outcomes.

*Muscle strength:* A dynamometer system (Humac Norm Systems, Stoughton, Massachusetts, USA) was used to measure the isometric peak torque (N/m) of the elbow flexors.^11^ The elbow flexor was tested at 60° of flexion as this joint angle has demonstrated the greatest elbow flexion torque in individuals with stroke.^12^ The test–retest reliability of the isometric elbow flexor test has been shown to be excellent (intra-class correlation coefficient (ICC)=0.97).^13^

*Sensory function:* The touch pressure threshold was tested using Semmes–Weinstein monofilament (SWMT).^14,15^ Both the dorsum of the hand (at the mid-length of the third proximal phalanges and mid-length of the third metacarpal bone) and the palmar side (i.e., the pulp of index finger and little finger, the midpoint of the thenar and hypothenar muscles) were tested. The greatest deficit in cutaneous sensation tends to occur at these sites among people with stroke.^14^ Light touch sensation (i.e., fingertip, palm and dorsum) was also significant correlated with upper extremity motor impairment scores (i.e., Wolf Motor Function Test and Fugl-Meyer Motor Assessment) (Spearman’s rank order correlation (r_s_)(40) = 0.31 to -0.45, p≤0.045).^14^ In individuals with chronic stroke, SWMT grade scores had substantial to perfect intra-rater agreement (thumb: Cohen’s kappa (κ)=0.89, index finger: κ=0.80) and substantial inter-rater agreement (thumb: κ=0.75, index finger: κ=0.79).^15^

*Physical activity level:* The Physical Activity Scale for the Elderly (PASE)^16^ was used to assess general physical activity (score range: 0–400). Twelve questions were asked regarding the frequency and duration of leisure activities, household activity, and work-related activity during the past week. The PASE has demonstrated good test–retest reliability (ICC=0.81).^16^

*Vascular health:* A Doppler ultrasound system (AixPlorer, Supersonic Imagine, Aix-en-Provence, France) was used in conjunction with a linear transducer (4-15MHz) to measure the blood flow velocity (cm/s), arterial diameter (cm) and blood flow volume (mL/min) of the brachial artery on each side.^17^ The settings of the Doppler ultrasound were standardized for high sensitivity, low wall filter with a pulsed repetition frequency of 7229 Hz. The sample volume was standardized at 0.5 mm. All participants were rested in a supine position for at least 15 minutes before the examination commenced. The room temperature was fixed at 23℃. The scanning of the two sides was conducted in a randomized order to minimize the order effect.

The participants were in a supine position throughout the ultrasound examination. The scan location of the brachial artery was standardized at the distal third point between the coracoid process and the crease of cubital fossa of the medial side.^18,19^ The elbow joint was standardized at 60° flexion using a custom arm immobilization device.

Three consistent spectral Doppler waveform cycle readings were used in one measurement. The baseline ultrasound scans were performed by an experienced researcher (Rater 1), and follow-up scan were performed by the first author (Rater 2). Each measurement was performed three times for both sides. Based on our pilot trial involving 15 individuals with chronic stroke, good to excellent inter-rater (ICC=0.87–0.92) and intra-rater reliability (ICC=0.92–0.98) were observed for peak systolic velocity and diameter of the brachial artery. Moderate inter-rater reliability (ICC=0.59–0.60) and good to excellent intra-rater reliability (ICC=0.82–0.93) were shown for blood flow volume of the brachial artery.

### ***Stroke-specific assessments***

Only participants in the stroke group underwent the following assessments.

*Motor Activity Log (MAL):* The MAL was used to measure the usage frequency and movement quality of the affected arm and hand during 30 daily activities.^20^ The test–retest correlation coefficient (Pearson’s r) was 0.91 for the quality of movement (QOM) and 0.50 for the amount of usage (AOU).

*Motor recovery:* The Fugl-Meyer Motor Assessment (Maximum: 66) was used to assess hemi-paretic upper limb motor recovery in terms of movement quality, movement coordination, and reflex action of the shoulder, elbow, forearm, wrist, and hand. This tool has previously shown excellent inter-rater reliability (ICC=0.96).^21^

*Spasticity:* Spasticity of the wrist/finger flexors was measured using the Composite Spasticity Scale (total score range: 1–16).^22^ This scale has previously shown excellent test–retest reliability (ICC=0.97).^22^

## **Supplementary Appendix 1.3**: Ultrasound protocol for brachial artery measures

The following steps were applied to obtain optimal imaging of the brachial artery:

*Step 1*: B-mode was applied in the beginning to find the artery with best image resolution. After marking the distal one-third of the upper arm, the probe was initially placed perpendicularly to the humerus to find the cross-sectional plane of the artery.

*Step 2*: Color Doppler was then used for visual confirmation of the artery. Thereafter, the probe was moved slightly medially or laterally to obtain the cross-sectional area in the middle of the visualization window, such that it was beneath the middle of the probe.

*Step 3*: The probe was rotated in the sagittal plane and tilted to be parallel with the artery. Fine adjustments were made to obtain an image with the longest continuity and the highest contrast between the Doppler blood flow signal and the artery wall.

*Step 4*: Within the visualization window, the electronic measurement calliper was then positioned at the center of the artery with the smallest insonation angle (i.e., the acute angle between the ultrasound beam and the blood flow direction within the artery).

*Step 5*: Pulse wave Doppler mode was then selected and the angle-to-flow was optimized at an insonation ≤ 60° by adjusting the Doppler steering and fine angle correction accordingly. The sample volume was standardized at 0.5 mm.

*Step 6*: Blood flow velocity (cm/s) and volume (mL/min) were then calculated from the spectral waveforms for each image captured. In order to detect the full range of positive and negative flow, the auto-trace function was applied. Arterial diameter (cm) was assessed using the electronic calliper to measure the distance between the superior and inferior endothelial boarders of the vessel lumen.

## **Supplementary Appendix 1.4**: Statistical analyses

First, the data in both the groups were summarized separately using descriptive statistics, as appropriate. Depending on whether the criteria for parametric statistics were fulfilled, Independent t-tests, Mann–Whitney U tests or chi-square tests were used to assess between-group differences in demographic variables.

Second, for each bone outcome and clinical outcomes with two sides (e.g., muscle strength, sensory, and vascular ultrasound measures), generalized estimating equation analysis [within-subject factors: side (2 levels: [stroke group] hemi-paretic vs. non-paretic; [healthy controls] dominant vs. non-dominant); time (2 levels: baseline vs. 2-year follow-up) and between-subject factor: group (2 levels: stroke vs. control)] with adjusted covariates (i.e., age, gender) were used to detect whether there was an interaction effect between these three factors. For variables demonstrating a significant three-way interaction, two separate generalized estimating equation analyses with the same adjusted covariates were performed to detect whether there were interaction effects between these two factors. The first analyzed the two within-subject factors: side (2 levels: stroke: hemi-paretic vs. non-paretic; healthy: dominant vs. non-dominant) and time (2 levels: baseline vs. 2-year follow-up)]. The second analyzed the between-subject factor: group (2 levels: stroke vs. control) and time (2 levels: baseline vs. 2-year follow-up). GEE is considered a regression method for correlated outcomes and has been used to address regression in the presence of dependent data, providing consistent coefficient estimates even with unspecified correlation structures (e.g., repeated-measures design).^23^

Third, we were interested in determining which aspects of bone (e.g., densitometric, microstructure, and cortical vs. trabecular) contributed most to the relative change in estimated failure load. To that end, we first used Pearson’s correlations to assess the relationship between the change in estimated failure load and the changes in other bone variables in the stroke group. Next, bone variables that showed significant correlations with the relative change in estimated failure load (p≤0.1) were entered into a multivariate regression model to identify their associations with the change in estimated failure load. To avoid multicollinearity, independent variables with a variance inflation factor (VIF) above 10 or strong intercorrelations (r>0.7)^24^ were eliminated from the prediction model. Akaike Information Criterion (AIC) was used as a comparative model performance metric, with lower values indicating better goodness of fit.^25^

Fourth, a second hierarchical multiple regression analysis was used to identify which clinical variables assessed at baseline were predictive of the relative change in estimated failure load. Likewise, bivariate correlations between the change in estimated failure load on the paretic side in the stroke group (i.e., dependent variables) at 2-year follow-up and potential influencing clinical variables (e.g., muscle strength, spasticity, and upper arm disuse) measured at baseline were examined. Variables showing significant correlation with the dependent variable (p<0.1) were selected as the independent predictors and entered into subsequent hierarchical multivariate regression analyses, while adjusting for potentially confounding factors (i.e., demographic characteristics). As many demographic factors (e.g., age, sex, and stroke duration) were measured, these were entered into a principal component analysis (i.e., confirmatory factor reduction). Factors extracted from the principal component analysis were first entered into the regression model, followed by the clinical variables identified above.^19^

Lastly, a third multivariate regression analysis was used to examine associations between the *changes* in clinical variables and the change in estimated failure load during the 2-year follow-up period, after accounting for the demographic factors mentioned above. The relative change values of all clinical outcome measures, with the exception of hand sensory function, were calculated using the following formula [(Baseline (T1) − 2-year follow-up (T2))/T1]. As sensory threshold does not have a zero point, it was not appropriate to use the relative change value for determining the decline in hand sensory function over time. Therefore, the absolute change value (T1 − T2) was used instead. Clinical variables were selected following the same principles used in selecting independent variables described above. In order to adjust for potential variation in scan region location, the percent change in participant height was added to the three prediction models to confirm whether the results remained stable. To assess linearity, error of variance and outlier effects in the regression analyses, homoscedasticity and distribution of residuals were determined by residual scatter plots and multivariate normality was determined by assessing the P-P plots of the residuals. In addition, a standardized residuals threshold was set between -3.3 to 3.3 to detect the presence of potential outliers for residuals falling outside this range.^24^

# **References**

1. Lam F, Bui M, Yang F, Pang M. Chronic effects of stroke on hip bone density and tibial morphology: a longitudinal study. *Osteoporosis international*. 2016;27(2):591-603.

2. Pang M, Zhang M, Li L, Jones A. Changes in bone density and geometry of the radius in chronic stroke and related factors: A one-year prospective study. *J Musculoskelet Neuronal Interact*. 2013;13(1):77-88.

3. Borschmann K, Iuliano S, Ghasem-Zadeh A, Pang MYC, Bernhardt J. The influence of physical activity and lean mass on volumetric bone density at the tibia two years after stroke. 2016:

4. Zhu TY, Griffith JF, Qin L, et al. Structure and strength of the distal radius in female patients with rheumatoid arthritis: A case-control study. *Journal of Bone and Mineral Research*. 2013/03/18 2013;28(4):794-806. doi:10.1002/jbmr.1793

5. Boutroy S, Bouxsein ML, Munoz F, Delmas PD. In VivoAssessment of Trabecular Bone Microarchitecture by High-Resolution Peripheral Quantitative Computed Tomography. *The Journal of Clinical Endocrinology & Metabolism*. 2005/12 2005;90(12):6508-6515. doi:10.1210/jc.2005-1258

6. Buie HR, Campbell GM, Klinck RJ, MacNeil JA, Boyd SK. Automatic segmentation of cortical and trabecular compartments based on a dual threshold technique for in vivo micro-CT bone analysis. *Bone*. 2007/10 2007;41(4):505-515. doi:10.1016/j.bone.2007.07.007

7. Burghardt AJ, Kazakia GJ, Ramachandran S, Link TM, Majumdar S. Age and Gender Related Differences in the Geometric Properties and Biomechanical Significance of Intra-Cortical Porosity in the Distal Radius and Tibia. *Journal of Bone and Mineral Research*. 2009/11/05 2009:091105035637074-32. doi:10.1359/jbmr.091104

8. Boutroy S, Van Rietbergen B, Sornay-Rendu E, Munoz F, Bouxsein ML, Delmas PD. Finite element analysis based on in vivo HR-pQCT images of the distal radius is associated with wrist fracture in postmenopausal women. *J Bone Miner Res*. Mar 2008;23(3):392-9. doi:10.1359/jbmr.071108

9. Pistoia W, van Rietbergen B, Lochmüller EM, Lill CA, Eckstein F, Rüegsegger P. Estimation of distal radius failure load with micro-finite element analysis models based on three-dimensional peripheral quantitative computed tomography images. *Bone*. Jun 2002;30(6):842-8. doi:10.1016/s8756-3282(02)00736-6

10. Zhu TY, Yip BH, Hung VW, et al. Normative Standards for HRpQCT Parameters in Chinese Men and Women. *J Bone Miner Res*. Oct 2018;33(10):1889-1899. doi:10.1002/jbmr.3481

11. Bohannon RW. Isokinetic testing of muscle strength of older individuals post-stroke: An integrative review. *Isokinetics and Exercise Science*. 2020;28:303-316. doi:10.3233/IES-201146

12. Ada L, Canning CG, Low SL. Stroke patients have selective muscle weakness in shortened range. *Brain*. Mar 2003;126(Pt 3):724-31. doi:10.1093/brain/awg066

13. Ekstrand E, Lexell J, Brogardh C. Isometric and isokinetic muscle strength in the upper extremity can be reliably measured in persons with chronic stroke. *J Rehabil Med*. Sep 2015;47(8):706-13. doi:10.2340/16501977-1990

14. Bowden JL, Lin GG, McNulty PA. The Prevalence and Magnitude of Impaired Cutaneous Sensation across the Hand in the Chronic Period Post-Stroke. *PloS one*. 2014/08/14 2014;9(8):e104153. doi:10.1371/journal.pone.0104153

15. Suda M, Kawakami M, Okuyama K, et al. Validity and Reliability of the Semmes-Weinstein Monofilament Test and the Thumb Localizing Test in Patients With Stroke. *Front Neurol*. 2020;11:625917. doi:10.3389/fneur.2020.625917

16. Ngai S, Cheung R, Lam P, Chiu J, Fung E. Validation and reliability of the Physical Activity Scale for the Elderly in Chinese population. *Journal of rehabilitation medicine*. 2012;44(5):462-465. doi:10.2340/16501977-0953

17. Chesbro SB, Asongwed ET, Brown J, John EB. Reliability of Doppler and Stethoscope Methods of Determining Systolic Blood Pressures: Considerations for Calculating an Ankle-Brachial Index. *Journal of the National Medical Association*. 2011/09 2011;103(9-10):863-869. doi:10.1016/s0027-9684(15)30441-7

18. Wu C-H, Ho Y-C, Hsiao M-Y, Chen W-S, Wang T-G. Evaluation of Post-Stroke Spastic Muscle Stiffness Using Shear Wave Ultrasound Elastography. *Ultrasound in Medicine & Biology*. 2017/06/01/ 2017;43(6):1105-1111. doi:<https://doi.org/10.1016/j.ultrasmedbio.2016.12.008>

19. Miller T, Ying MTC, Hung VWY, et al. Determinants of estimated failure load in the distal radius after stroke: An HR-pQCT study. *Bone*. 2020/12/23/ 2020:115831. doi:<https://doi.org/10.1016/j.bone.2020.115831>

20. van der Lee JH, Beckerman H, Knol DL, de Vet HCW, Bouter LM. Clinimetric Properties of the Motor Activity Log for the Assessment of Arm Use in Hemiparetic Patients. *Stroke*. 2004/06 2004;35(6):1410-1414. doi:10.1161/01.str.0000126900.24964.7e

21. Sanford J, Moreland J, Swanson LR, Stratford PW, Gowland C. Reliability of the Fugl-Meyer assessment for testing motor performance in patients following stroke. Article. *Physical Therapy*. 1993;73(7):447-454. doi:10.1093/ptj/73.7.447

22. Ng SS, Hui-Chan CW. The Timed Up & Go Test: Its Reliability and Association With Lower-Limb Impairments and Locomotor Capacities in People With Chronic Stroke. *Archives of Physical Medicine and Rehabilitation*. 2005/08 2005;86(8):1641-1647. doi:10.1016/j.apmr.2005.01.011

23. Liang K-Y, Zeger SL. Longitudinal data analysis using generalized linear models. *Biometrika*. 1986;73(1):13-22. doi:10.1093/biomet/73.1.13

24. Tabachnick B, Fidell L. *Using Multivariate Statistics*. Pearson Education; 2018.

25. Akaike H. A new look at the statistical model identification. *IEEE Transactions on Automatic Control*. 1974;19(6):716-723. doi:10.1109/TAC.1974.1100705
